# Supplementary material for: A UMLS-based spell checker for natural language processing in vaccine safety
Source: BMC Med Inform Decis Mak. 2007 Feb 12;7:3. doi: 10.1186/1472-6947-7-3 (PMC1805499; doi:10.1186/1472-6947-7-3)
Supplement: Additional file 13 — RAP application directory. Sets up directory for RAP (RDF application for PHP) source code [file 1472-6947-7-3-S13.gz › rap/doc/phpdoc/__filesource/fsource_default__vocabularyATOM_RES.php.html]

xml version="1.0" encoding="iso-8859-1"?


File Source for ATOM\_RES.php


# Source for file ATOM\_RES.php

Documentation is available at ATOM\_RES.php

```
1. <?PHP
2. // ----------------------------------------------------------------------------------
3. // ATOM Vocabulary (ResResource)
4. // ----------------------------------------------------------------------------------
5. // Version                   : 0.9
6. // Authors                   : Tobias Gau� (tobias.gauss@web.de)
7. //
8. // Description               : Wrapper, defining resources for all terms of the
9. //                               ATOM Vocabulary;.
10. //                               For details about ATOM see: http://semtext.org/atom/atom.html.
11. //                                Using the wrapper allows you to define all aspects of
12. //                             the vocabulary in one spot, simplifing implementation and
13. //                             maintainence.
14. //
15. // ----------------------------------------------------------------------------------
16. // History:
17. // 11-08-2003                 : Initial version
18. // ----------------------------------------------------------------------------------


24. class ATOM_RES{

26. function TYPE()
27. {
28. return  new ResResource(ATOM_NS . 'type');

30. }

32. function MODE()
33. {
34. return  new ResResource(ATOM_NS . 'mode');

36. }

38. function NAME()
39. {
40. return  new ResResource(ATOM_NS . 'name');

42. }

44. function URL()
45. {
46. return  new ResResource(ATOM_NS . 'url');

48. }

50. function EMAIL()
51. {
52. return  new ResResource(ATOM_NS . 'email');

54. }

56. function REL()
57. {
58. return  new ResResource(ATOM_NS . 'rel');

60. }

62. function HREF()
63. {
64. return  new ResResource(ATOM_NS . 'href');

66. }

68. function TITLE()
69. {
70. return  new ResResource(ATOM_NS . 'title');

72. }

74. function ATOM_CONSTRUCT()
75. {
76. return  new ResResource(ATOM_NS . 'AtomConstruct');

78. }

80. function CONTENT()
81. {
82. return  new ResResource(ATOM_NS . 'Content');

84. }

86. function PERSON()
87. {
88. return  new ResResource(ATOM_NS . 'Person');

90. }

92. function VALUE()
93. {
94. return  new ResResource(ATOM_NS . 'value');

96. }

98. function LINK()
99. {
100. return  new ResResource(ATOM_NS . 'Link');

102. }

104. function FEED()
105. {
106. return  new ResResource(ATOM_NS . 'Feed');

108. }

110. function VERSION()
111. {
112. return  new ResResource(ATOM_NS . 'version');

114. }

116. function LANG()
117. {
118. return  new ResResource(ATOM_NS . 'lang');

120. }

122. function AUTHOR()
123. {
124. return  new ResResource(ATOM_NS . 'author');

126. }

128. function CONTRIBUTOR()
129. {
130. return  new ResResource(ATOM_NS . 'contributor');

132. }

134. function TAGLINE()
135. {
136. return  new ResResource(ATOM_NS . 'tagline');

138. }

140. function GENERATOR()
141. {
142. return  new ResResource(ATOM_NS . 'generator');

144. }

146. function COPYRIGHT()
147. {
148. return  new ResResource(ATOM_NS . 'copyright');

150. }

152. function INFO()
153. {
154. return  new ResResource(ATOM_NS . 'info');

156. }

158. function MODIFIED()
159. {
160. return  new ResResource(ATOM_NS . 'modified');

162. }

164. function ENTRY()
165. {
166. return  new ResResource(ATOM_NS . 'Entry');

168. }

170. function HAS_CHILD()
171. {
172. return  new ResResource(ATOM_NS . 'hasChild');

174. }

176. function HAS_ENTRY()
177. {
178. return  new ResResource(ATOM_NS . 'hasEntry');

180. }

182. function HAS_LINK()
183. {
184. return  new ResResource(ATOM_NS . 'hasLink');

186. }

188. function HAS_TITLE()
189. {
190. return  new ResResource(ATOM_NS . 'hasTitle');

192. }

194. function ISSUED()
195. {
196. return  new ResResource(ATOM_NS . 'issued');

198. }

200. function CREATED()
201. {
202. return  new ResResource(ATOM_NS . 'created');

204. }

206. function SUMMARY()
207. {
208. return  new ResResource(ATOM_NS . 'summary');
209. }
210. }


214. ?>
```

Documentation generated on Fri, 17 Dec 2004 16:14:23 +0100 by phpDocumentor 1.3.0RC3
